# Evaluating Abdominal Obesity and BMI As Risk Factors for Diabetic Nephropathy in Patients With Type 2 Diabetes Mellitus: A Case-Control Study

**DOI:** 10.7759/cureus.92085

**Published:** 2025-09-11

**Authors:** Safiyyah Ubaid, Muhammad Osama, Ubaid Ullah, Zahir Ud Din, Muhammad Zohaib Ul Hassan, Muhammad Hamza

**Affiliations:** 1 Biochemistry, Khyber Medical College, Peshawar, PAK; 2 Internal Medicine, Hayatabad Medical Complex Peshawar, Peshawar, PAK; 3 Internal Medicine, Khyber Medical College, Peshawar, PAK; 4 Radiology, Khyber Medical College, Peshawar, PAK

**Keywords:** bmi, diabetic nephropathy, dyslipidemia, type 2 diabetes mellitus, waist circumference

## Abstract

Introduction

Diabetic nephropathy (DN) is a major microvascular complication of type 2 diabetes, contributing significantly to chronic kidney disease (CKD) and end-stage renal failure. Obesity, particularly central obesity, has emerged as a potential risk factor; however, its role in DN remains uncertain. This study aimed to determine the association of central obesity and BMI with DN in patients with type 2 diabetes mellitus. It also sought to evaluate the role of waist circumference (WC), HbA1c, and lipid profile as potential metabolic predictors of nephropathy risk.

Material and methods

A case-control study was conducted from March to September 2024 in Peshawar, involving 98 cases and 49 controls. Patients were matched for age, and data were collected on BMI, HbA1c, WC, and proteinuria. Statistical analyses included correlation, regression analysis, and Chi-square tests using IBM SPSS version 20.

Results

The study included 98 cases and 49 controls, with a mean age of 55.9 ± 13.2 years. The gender distribution was 56% female in the case group and 49% in the control group. Chi-square analysis revealed significant associations between DN and WC (OR = 2.71), duration of diabetes (OR = 2.78), BMI, HbA1c, oral antidiabetic medication use, and dyslipidemia. Proteinuria showed a moderate, significant positive correlation with BMI (r = 0.402), HbA1c (r = 0.432), and low-density lipoprotein (LDL, r = 0.242). A weak but significant positive correlation was observed with WC (r = 0.231), triglycerides (r = 0.197), and total cholesterol (r = 0.221). Regression analysis identified BMI, HbA1c, and duration of diabetes as significant risk factors.

Conclusion

Abdominal obesity, as indicated by WC, was significantly associated with DN in patients with type 2 diabetes mellitus. BMI, HbA1c, and diabetes duration were key risk factors, highlighting the need for comprehensive metabolic management in assessing DN risk.

## Introduction

Obesity is a global problem and is increasingly common in many countries. A large multicentric study across 288 population-based studies involving 13,233,675 individuals reported the global prevalence of central obesity to be 41.5% (95% CI: 39.9-43.2%) [1]. General obesity is defined as a BMI of 30 kg/m² or greater, indicating excessive body fat based on weight and height. A waist circumference (WC) of 102 cm or greater for men and 88 cm or greater for women reflects excess abdominal fat accumulation. Obesity is usually classified as general or central (abdominal) [2]. As part of the metabolic syndrome, obesity increases the risk of a wide range of chronic diseases. Increasing evidence suggests that obesity is a risk factor for diabetes and chronic kidney disease. As markers of obesity, high BMI, WC, and waist-to-hip ratio have been reported to be related to diabetic nephropathy (DN) and end-stage renal disease [3].

Some studies show that central obesity is strongly associated with DN and other kidney diseases [4]. A meta-analysis conducted by Zhao Q et al. found that WC was significantly associated with adverse renal outcomes in diabetic patients [5]. On the contrary, a study conducted by Kanakamani J et al. showed no association between WC and DN [6].

A study conducted by Mohammedi K et al. demonstrated that in patients with a BMI above 25 kg/m², the risk of major renal events in diabetic patients increased progressively as BMI increased [7]. Abdominal obesity, rather than general obesity, was more closely associated with DN, as shown in a study by Wan H et al. [8]. However, an inverse relationship between BMI and diabetic kidney events has also been observed [9,10]. One cohort study conducted by Bentata Y et al. reported that the decline of kidney function in diabetic patients was not directly influenced by BMI, and end-stage renal disease was observed in normal-weight, overweight, and obese patients alike [11].

Therefore, there is ambiguity regarding the relationship between WC, BMI, and the development of DN. The present study aims to clarify this relationship among patients with type 2 diabetes mellitus.

## Materials and methods

Study design and settings

This was a case-control study conducted from March 2024 to September 2024 in the tertiary care hospital of Peshawar, Khyber Teaching Hospital (KTH), involving two departments: the Department of General Medicine and the Department of Endocrinology.

Sample size

The sample size was calculated using Open-Epi, Version 3, with a CI of 95%, power of 90%, a case-to-control ratio of 1:0.5, and an odds ratio (OR) of 1.4, based on a similar study. The calculated sample size was 98 cases and 49 controls, resulting in a total of 147 participants. The sampling technique used was simple random sampling [12].

Inclusion and exclusion criteria

Cases were defined as adult individuals of both genders diagnosed with type II diabetes mellitus who exhibited DN, according to the operational definition (a single positive proteinuria result detected using a dipstick test in the presence of retinopathy and absence of any other kidney disease). According to Kidney Disease: Improving Global Outcomes (KDIGO), “diabetic nephropathy is a form of CKD persisting for more than three months, characterized by an estimated glomerular filtration rate (eGFR) below 60 mL/min/1.73 m² and/or evidence of kidney damage, such as an elevated urinary albumin-to-creatinine ratio (UACR) ≥30 mg/g, often presenting as proteinuria.” Controls were defined as adult individuals of both genders diagnosed with type II diabetes mellitus but without DN. DN was ruled out by a negative dipstick test for proteinuria or evidence of any other kidney disease. Exclusion criteria included individuals with type I diabetes mellitus and those with renal diseases unrelated to DN.

Data collection procedure

Patients were selected from the hospital database based on the inclusion criteria and classified into cases and controls. Consent was obtained for the use of patient data, ensuring confidentiality and confirming that there was no direct risk to patients from participating in the study. All medical records used for this study were de-identified by removing personal identifiers such as patient name, registration number, and contact details. Only coded data were used for statistical purposes.

Cases were identified based on urinary protein levels (dipstick protein positivity as well as UACR ≥30 mg/g) and extracted from hospital records where available. DN (cases) was defined according to the operational definition: “a single positive proteinuria result using the dipstick test, accompanied by retinopathy and the absence of any other kidney disease.”

Baseline demographic and metabolic information of the selected cases were extracted from the hospital database as risk factors. Data collected included age, gender, duration of diabetes, BMI (weight measured in kilograms using a calibrated digital scale and height measured in meters using a stadiometer; BMI calculated as weight/height²), HbA1c, lipid profile, oral diabetic medications, and socioeconomic status. For abdominal obesity, WC was measured using standardized techniques (measured at the midpoint between the lower rib margin and iliac crest with a non-stretchable tape, following WHO guidelines).

Data analysis

Data were analyzed using IBM SPSS Statistics version 20. Frequencies and percentages were computed for qualitative variables such as DN. Mean ± SD was presented for quantitative variables such as BMI and HbA1c. DN was stratified among different WC and BMI groups. Post-stratification Chi-square test was applied. Correlation and multiple regression analyses were also performed. A p-value of ≤0.05 was considered statistically significant.

## Results

The study included 98 cases and 49 controls. The mean age was 55.9 ± 13.2 years and was nearly the same in both groups, with no statistically significant difference observed. Gender distribution was 56% female in the cases and 49% female in the controls, with no statistical difference between groups (Table 1). The mean BMI of the patients was 23.8 ± 3.7, while the mean HbA1c was 8.9 ± 2.4. The mean waist circumference was 35.3 ± 3.5 cm. Chi-square analysis showed that education level, duration of diabetes (OR 2.78), BMI, HbA1c, abdominal obesity (WC) (OR 2.71), oral antidiabetic medication use, and dyslipidemia, except for low HDL, were significantly associated with diabetic nephropathy (Table 1).

**Table 1:** Chi-square analysis of variables associated with proteinuria among diabetic patients. WC: Waist Circumference; LDL: Low-Density Lipoprotein; HDL: High-Density Lipoprotein.

Variables	Proteinuria		P-value	χ² value	OR	95% CI	
	Present	Absent				Lower	Upper
Age (years)			0.294	1.103	1.44	0.725	2.88
> 60	51	21					
≤ 60	47	28					
Gender			0.413	0.67	1.33	0.67	2.65
Female	55	24					
Male	43	25					
Education level			0.005	7.86	2.71	1.338	5.52
Below matriculation	60	18					
Above matriculation	38	31					
Residence			0.27	1.21	1.491	0.732	3.035
Rural	67	29					
Urban	31	20					
Duration of diabetes (years)			0.004	8.25	2.78	1.37	5.6
> 2.5	68	22					
≤ 2.5	30	27					
BMI (kg/m²)			<0.0001	24.3	N/A	N/A	N/A
< 25	21	30					
25-29.9	69	19					
≥ 30	8	0					
Waist circumference (cm)			0.005	7.86	2.71	1.33	5.52
> 35	60	18					
≤ 35	38	31					
HbA1c (%)			<0.0001	31.88	N/A	N/A	N/A
≤ 7.5	23	35					
7.6-10	39	9					
> 10	36	5					
Insulin use			0.476	0.507	0.774	0.382	1.567
Yes	56	31					
No	42	18					
Oral diabetic medicine			<0.0001	24.91	N/A	N/A	N/A
Glimepiride	7	8					
Linagliptin + Empagliflozin	9	18					
Sitagliptin + Metformin	15	6					
Metformin	56	16					
Vildagliptin + Metformin	9	0					
Metformin + Sitagliptin	2	1					
LDL (mg/dl)			0.003	8.61	2.84	1.4	5.7
> 100	63	19					
≤ 100	35	30					
Total cholesterol (mg/dl)			0.007	7.2	2.6	1.2	5.4
> 200	55	16					
≤ 200	43	33					
HDL (mg/dl)			0.245	1.35	0.62	0.283	1.385
≤ 40	67	38					
> 40	31	11					
Triglyceride (mg/dl)			0.01	5.69	2.33	1.15	4.7
> 150	66	23					
≤ 150	32	26					

Correlation analysis revealed significant relationships between proteinuria and several clinical factors. Proteinuria showed a moderate, significant positive correlation with BMI (r = 0.402), HbA1c (r = 0.432), and LDL (r = 0.242). A weak but significant positive correlation was observed with WC (r = 0.231), triglycerides (r = 0.197), and total cholesterol (r = 0.221). The correlation between proteinuria and IU was weak, negative, and non-significant (r = -0.059). These results highlight the importance of BMI, HbA1c, and LDL in relation to proteinuria, while other variables showed weaker associations (Table 2).

**Table 2:** Correlation analysis between proteinuria and various clinical variables. EL: Education level; WC: Waist circumference; TGs: Triglyceride; TC: Total cholesterol; DOM: Diabetic oral medication; DOD: Duration of diabetes; IU: Insulin use; LDL: Low-Density Lipoprotein; HDL: High-Density Lipoprotein.

Variables	EL	DOD	BMI	WC	HbA1c	IU	TGs	LDL	HDL	TC
Proteinuria	.231**	.237**	.402**	.231**	.432**	-0.059	.197*	.242**	-0.096	.221**
EL		-0.133	.238**	.344**	.202*	-0.032	-0.034	.206*	-0.142	0.091
DOD			0.008	0.105	0.059	-0.15	-0.043	-0.006	0.053	0.127
BMI				.287**	.283**	0.038	-0.149	-.317**	0.034	.358**
WC					.369**	-0.088	.189*	.288**	-0.112	.282**
HbA1c						-0.137	-0.159	-.194*	0.146	.205*
IU							-0.076	-0.071	-0.066	-0.056
TGs								0.122	-.202*	.446**
LDL									-0.139	.395**
HDL										-0.052
TC										
** Correlation is significant at the 0.01 level (2-tailed).										
* Correlation is significant at the 0.05 level (2-tailed).										

**Figure 1:**
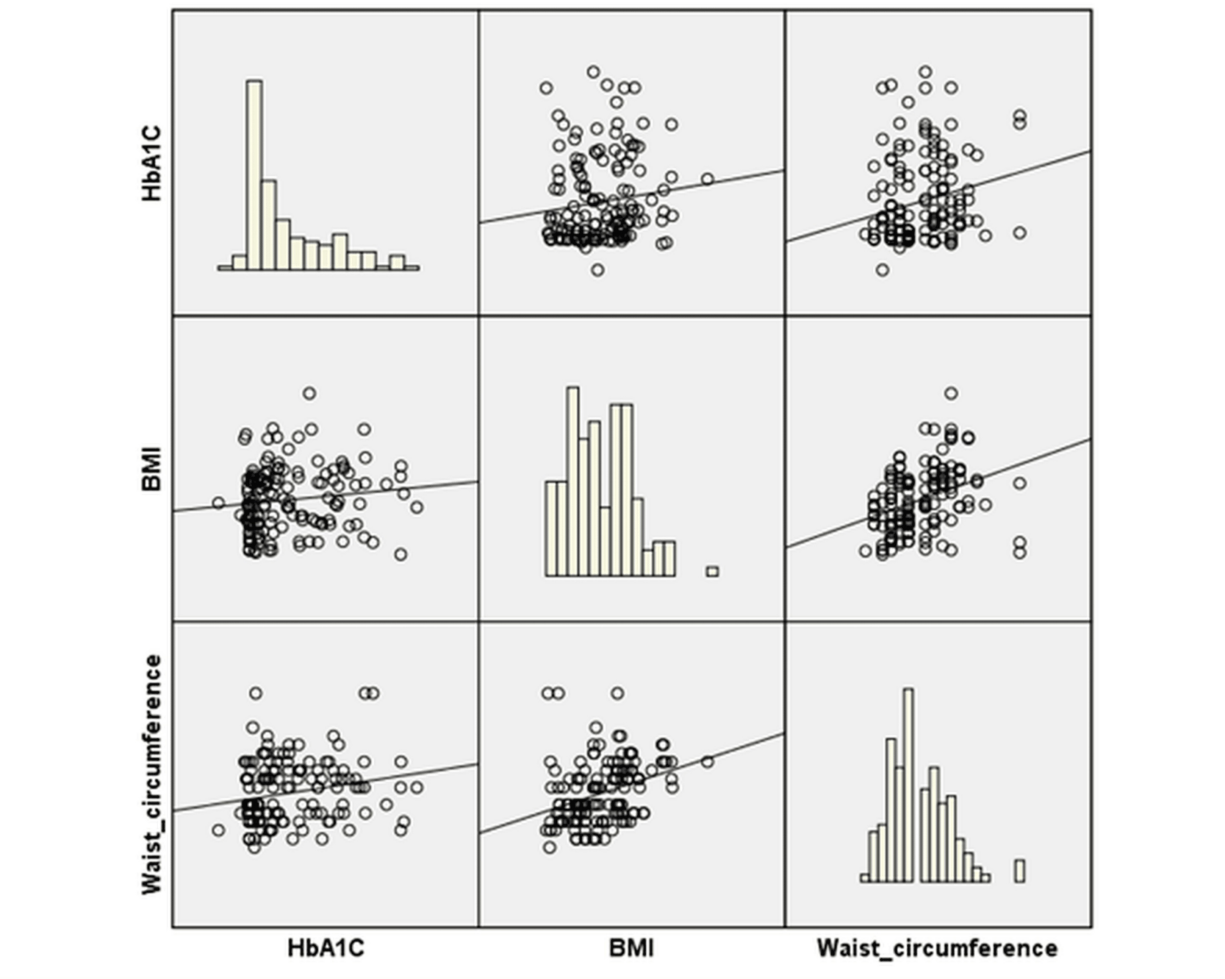
Scatter matrix plot and histograms showing the relationships and distributions of HbA1c, BMI, and waist circumference.

A scatter plot matrix was generated to demonstrate the correlations (Figure 1). The histograms suggested that while HbA1c and BMI had more skewed distributions, WC was closer to a normal distribution. These relationships are relevant, as higher BMI and WC were typically linked to poorer glycemic control (HbA1c), indicating a potential interconnection between obesity-related parameters and diabetes management (Figure 1).

The multiple regression model showed that BMI, HbA1c, and duration of diabetes among the independent variables had significant predictive value for the dependent variable, DN (p < 0.05). Moreover, the R² = 0.359 indicated that the model explained 35.9% of the variance in DN (Table 3).

**Table 3:** Multiple regression analysis of the dependent variable. LDL: Low-Density Lipoprotein; HDL: High-Density Lipoprotein.

Variables	Unstandardized Coefficients		Standardized Coefficients	t	Sig.	95% CI for B	
	B	Std. Error	Beta			Lower Bound	Upper Bound
Constant	1.328	0.385	–	3.447	0.001	0.566	2.09
Age	0.007	0.081	0.008	0.089	0.93	-0.152	0.167
Gender	0.015	0.079	0.016	0.185	0.853	-0.142	0.171
Education level	0.119	0.087	0.126	1.369	0.173	-0.053	0.291
Address (residence)	0.03	0.071	0.03	0.427	0.67	-0.109	0.17
Duration of diabetes (years)	0.236	0.077	0.244	3.062	0.003	0.084	0.389
BMI	-0.221	0.067	-0.264	-3.289	0.001	-0.354	-0.088
Waist circumference	-0.007	0.079	-0.007	-0.085	0.932	-0.163	0.15
HbA1c	-0.173	0.046	-0.298	-3.752	0	-0.264	-0.082
Insulin use	-0.037	0.07	-0.038	-0.524	0.601	-0.176	0.102
Triglycerides	0.132	0.083	0.137	1.594	0.113	-0.032	0.297
LDL	0.085	0.077	0.09	1.109	0.27	-0.067	0.238
HDL	-0.002	0.076	-0.002	-0.025	0.98	-0.153	0.149
Total cholesterol	-0.073	0.087	-0.077	-0.832	0.407	-0.245	0.1
a. Dependent Variable: Proteinuria							
R² = 0.359; Adjusted R² = 0.296; R = 0.599; Std. Error = 0.397							

## Discussion

This case-control study investigated abdominal obesity, BMI, and other metabolic factors as risk factors for diabetic nephropathy in type 2 diabetes mellitus. Our findings revealed significant associations between diabetic nephropathy and several factors, including WC, gender, HbA1c, BMI, oral antidiabetic medications, and dyslipidemia. Although abdominal obesity was significantly associated with diabetic nephropathy, it was not a significant predictor in regression analysis. These results highlight the importance of comprehensive metabolic management in assessing nephropathy risk in diabetic patients.

In the ADVANCE study, each additional unit of BMI above 25 kg/m² was associated with a 4% (ranging from 1% to 6%) increase in the risk of major renal events [7]. This is similar to our results, in which BMI showed a moderate, significant positive association with diabetic nephropathy.

Among various indices of abdominal obesity, the Chinese Visceral Adiposity Index (CVAI) showed the strongest association with the prevalence of diabetic kidney disease. For men, each standard deviation increase in CVAI was linked to a 38% higher likelihood of diabetic kidney disease (OR 1.38; 95% CI 1.12-1.70). In women, each standard deviation increase in CVAI corresponded to a significantly higher prevalence, with an odds ratio of 2.50 (95% CI 1.81-3.47) [8], showing that abdominal obesity rather than general obesity is closely associated with diabetic nephropathy. In our study, abdominal obesity (WC) (r = 0.231, p < 0.01) also exhibited positive correlations with proteinuria, indicating significant associations with higher waist measurements. In contrast, however, a 5-year prospective analysis of the Hong Kong Diabetes Registry and another study suggested an inverse relationship [9,10].

The slowest decline in kidney function was linked to the lowest baseline HbA1c levels, with CKD manifestation taking 15.9 years for HbA1c levels below 5.7% and 8.3 years for HbA1c >8.5% [13]. HbA1c also had significant predictive value for diabetic nephropathy in our study. A risk threshold for HbA1c linked to albuminuria was established in a Chinese population over 40 years of age. The albumin-to-creatinine ratio was positively and independently correlated with HbA1c ≥5.5% [14]. These findings imply that early blood glucose management and renal function testing are essential for preventing diabetic kidney disease in at-risk populations. In our study, a strong positive correlation was found between HbA1c levels and proteinuria. The odds ratio of microalbuminuria increased with higher HbA1c levels, and the risk for macroalbuminuria also rose at higher HbA1c levels [15]. Our study further confirmed a significant association between HbA1c and diabetic nephropathy, with HbA1c as an independent predictor. Glucose regulation and setting a target to maintain HbA1c within the recommended range in diabetic patients may help reduce the risk of proteinuria [16].

In a longitudinal cohort study conducted in the US, with a mean age of 51.1 years and 70% male participants, remnant cholesterol levels were used to assess the potential for developing nephropathy in diabetic patients, as it was a less expensive and easily calculated parameter. The study found a positive association between remnant cholesterol levels and the development of end-stage kidney disease in patients with type 2 diabetes mellitus. In contrast, our study, which had a higher mean age of 55.9 years and nearly half male participants, utilized a full lipid profile. We observed that all lipid parameters, except HDL, were significant risk factors for diabetic nephropathy [17].

One study showed that the DN patient group had a significant decline in HDL values, while their triglyceride values increased significantly compared to the control group. A decline in cholesterol and LDL levels was insignificant [18]. In a review of seven studies from various countries, decreased GFR and albuminuria were associated with greater TG and LDL variability, but lower HDL levels appeared to protect against microalbuminuria [19]. The protective effect of low LDL is in contrast to our study. Dyslipidemia contributes to kidney damage and worsening renal function in patients with diabetes. Specifically, elevated levels of LDL cholesterol and triglycerides are associated with an increased risk of kidney complications [20]. These results are consistent with our study.

Our study also showed a significant association of oral antidiabetic drugs with diabetic nephropathy (Linagliptin + Empagliflozin reduced the risk of DN). There is limited comparative evidence regarding the impact of various treatments for type 2 diabetes on renal outcomes, whether used alone or in combination [21]. According to prospective cohort research, metformin significantly lowers all-cause mortality in people with type 2 diabetes, indicating that treatment has advantages beyond glucose management [22]. In people with type 2 diabetes, taking SGLT2 inhibitors or GLP-1 receptor agonists was linked to a lower risk of serious kidney issues compared to DPP-4 inhibitors or sulfonylureas, which aligns with our findings [23]. Starting treatment with dapagliflozin, a type of SGLT2 inhibitor, helped reduce urinary protein levels (albuminuria) in patients with type 2 diabetes who were at low risk of kidney complications [24]. According to a review of several studies, DPP-4 inhibitors helped maintain kidney function in people with diabetes and reduced markers of kidney damage [25].

Limitations

One limitation of this case-control study is the reliance on hospital records and a relatively small sample size. Additionally, unmeasured confounding factors, such as lifestyle and dietary habits, may have affected the associations between risk factors and diabetic nephropathy. Selection bias may also have arisen, as cases and controls were drawn from a hospital setting, which might not represent the broader diabetic population.

## Conclusions

In conclusion, this case-control study identified several significant risk factors for DN. Abdominal obesity, as indicated by WC, was significantly associated with proteinuria, highlighting the role of central obesity in disease progression. Glycemic control (HbA1c), dyslipidemia, and oral antidiabetic medication use were also significantly associated with nephropathy risk. Overall, the study underscores the importance of managing metabolic and lifestyle factors to reduce the risk of DN.

## Appendices

Appendix 1

**Table 4:** STROBE checklist. STROBE: Strengthening the Reporting of Observational Studies in Epidemiology.

	Item No	Recommendation	Page No
Title and abstract	1	(a) Indicate the study’s design with a commonly used term in the title or the abstract	1
		(b) Provide in the abstract an informative and balanced summary of what was done and what was found	2,3
Introduction			
Background/rationale	2	Explain the scientific background and rationale for the investigation being reported	3,4
Objectives	3	State specific objectives, including any prespecified hypotheses	4
Methods			
Study design	4	Present key elements of study design early in the paper	4
Setting	5	Describe the setting, locations, and relevant dates, including periods of recruitment, exposure, follow-up, and data collection	4
Participants	6	(a) Cohort study-Give the eligibility criteria, and the sources and methods of selection of participants. Describe methods of follow-up Case-control study-Give the eligibility criteria, and the sources and methods of case ascertainment and control selection. Give the rationale for the choice of cases and controls Cross-sectional study-Give the eligibility criteria, and the sources and methods of selection of participants	5
		(b) Cohort study-For matched studies, give matching criteria and number of exposed and unexposed Case-control study-For matched studies, give matching criteria and the number of controls per case	4
Variables	7	Clearly define all outcomes, exposures, predictors, potential confounders, and effect modifiers. Give diagnostic criteria, if applicable	4, 5
Data sources/ measurement	8*	For each variable of interest, give sources of data and details of methods of assessment (measurement). Describe comparability of assessment methods if there is more than one group	5
Bias	9	Describe any efforts to address potential sources of bias	4,5
Study size	10	Explain how the study size was arrived at	4
Quantitative variables	11	Explain how quantitative variables were handled in the analyses. If applicable, describe which groupings were chosen and why	6
Statistical methods	12	(a) Describe all statistical methods, including those used to control for confounding	6
		(b) Describe any methods used to examine subgroups and interactions	
		(c) Explain how missing data were addressed	
		(d) Cohort study-If applicable, explain how loss to follow-up was addressed Case-control study-If applicable, explain how matching of cases and controls was addressed Cross-sectional study-If applicable, describe analytical methods taking account of sampling strategy	4
		(e) Describe any sensitivity analyses	
